# Ruxolitinib prevents irreversible autoimmune endocrinopathies in APECED

**DOI:** 10.1038/s41467-026-76285-x

**Published:** 2026-08-01

**Authors:** Joseph Pechacek, Taura Webb, Lucas dos Santos Dias, Rachel Wu, Heather Moorman, Princess Barber, Lindsey B. Rosen, Peter D. Burbelo, Benjamin Colton, Herodes Guzman, Maria Sacta, Shana E. McCormack, Aaron Bennett, Kathleen Loomes, Neil Romberg, Niamh McGrath, Steven M. Holland, Theo Heller, Karen K. Winer, Stefania Pittaluga, Michail S. Lionakis

**Affiliations:** 1 Fungal Pathogenesis Section, Laboratory of Clinical Immunology and Microbiology (LCIM), National Institute of Allergy and Infectious Diseases (NIAID), National Institutes of Health (NIH), Bethesda, MD USA; 2 Laboratory of Pathology, National Cancer Institute, NIH, Bethesda, MD USA; 3 Immunopathogenesis Section, LCIM, NIAID, NIH, Bethesda, MD USA; 4 Adeno-Associated Virus Biology Section, National Institute of Dental and Craniofacial Research (NIDCR), NIH, Bethesda, Maryland USA; 5 Pharmacy Department, NIH Clinical Center, Bethesda, Maryland USA; 6 Division of Endocrinology and Diabetes, Children’s Hospital of Philadelphia, Philadelphia, PA USA; 7 Division of Human Genetics, Children’s Hospital of Philadelphia, Philadelphia, PA USA; 8 Division of Allergy and Immunology, Children’s Hospital of Philadelphia, Philadelphia, PA USA; 9 Division of Gastroenterology, Hepatology, and Nutrition, Children’s Hospital of Philadelphia, Philadelphia, PA USA; 10 School of Public Health, University College Dublin, Dublin, Ireland; 11 Liver Diseases Branch, National Institute of Diabetes and Digestive and Kidney Diseases, NIH, Bethesda, MD USA; 12 Pediatric Growth and Nutrition Branch, National Institute of Child Health and Human Development, NIH, Bethesda, MD USA

**Keywords:** Translational research, Autoimmunity

## Abstract

Autoimmune polyendocrinopathy-candidiasis-ectodermal dystrophy (APECED/APS-1) is a monogenic disorder of failed central tolerance. Interferon-γ-driven inflammation in APECED underlies multiorgan autoimmunity and chronic mucocutaneous candidiasis and is targetable with JAK1/2 inhibition. Whether early cytokine blockade can avert irreversible endocrine failure is currently unknown. Here, we describe two individuals with APECED who developed evolving autoimmune hypoparathyroidism and hypergonadotropic hypogonadism. Treatment with the JAK1/2 inhibitor ruxolitinib halted progression and reversed biochemical and clinical abnormalities, preserving parathyroid and gonadal function. These findings provide clinical evidence that timely JAK-STAT pathway inhibition can intercept evolving endocrine autoimmunity in APECED. More broadly, they advance a disease-interception paradigm in which early, pathway-directed cytokine blockade may alter the natural history of autoimmune endocrinopathies.

## Abstract (web summary)

APECED is a monogenic autoimmune disorder resulting from deleterious variants in the Autoimmune Regulator gene. Here, the authors report the cases of two patients with APECED in whom early intervention with the JAK1/2 inhibitor ruxolitinib prevented endocrine autoimmunity.

## Introduction

Autoimmune PolyEndocrinopathy-Candidiasis-Ectodermal Dystrophy (APECED; also known as Autoimmune Polyendocrine Syndrome type-1, APS-1) is a monogenic disorder of impaired central tolerance that causes substantial morbidity and mortality^1^. APECED results from deleterious variants in the Autoimmune Regulator (*AIRE*) gene, which disrupts thymic negative selection of autoreactive T cells, permitting their escape into the periphery and leading to early-onset multiorgan autoimmunity and chronic mucocutaneous candidiasis (CMC)^2,3^. Beyond the classic triad manifestations of CMC, adrenal insufficiency, and hypoparathyroidism, patients develop dysfunction of multiple non-endocrine and endocrine organs, the latter culminating in irreversible, life-threatening endocrinopathies that require lifelong hormone replacement^1,4–6^.

We recently showed that excessive interferon-γ (IFN-γ) is a dominant driver of autoimmune tissue injury and CMC in APECED^7,8^, and that inhibition of Janus kinases 1/2 (JAK1/2) with ruxolitinib ameliorates autoimmune and fungal infection manifestations in both Aire-deficient mice and patients with APECED^8–12^. Mechanistically, IFN-γ signals through JAK1/2 to phosphorylate STAT1 (signal transducer and activator of transcription 1), leading to transcription of IFN-γ–responsive genes, including the chemokine CXCL9, a surrogate marker of pathway activation. Thus, JAK inhibition may dampen the IFN-γ–driven inflammatory program in APECED and mitigate tissue injury. However, whether early IFN-γ pathway blockade can intercept evolving autoimmune endocrinopathies before irreversible glandular destruction occurs remains unknown.

Effective disease-modifying therapies for autoimmune endocrine failure remain rare. Teplizumab, an anti-CD3 monoclonal antibody that delays progression to stage-3 type-1 diabetes, represents a notable example of disease interception in endocrine autoimmunity^13,14^. Here, we describe two patients with APECED in whom early initiation of ruxolitinib stabilized and reversed evolving hypoparathyroidism and hypogonadism. These findings illustrate a disease-interception model in which early, pathway-directed cytokine blockade alters the clinical trajectory of autoimmune endocrinopathies.

## Results

### Patient 1

A two-year-old boy with APECED (carrying compound heterozygous *AIRE* variants c.2 T > A and c.967_979del) presented with autoimmune hepatitis, CMC, and nail dystrophy. At 17 months of age, he developed markedly elevated aminotransferases (AST 3191 U/L; ALT 2574 U/L) and positive anti-liver-kidney microsomal antibodies. Liver biopsy confirmed APECED-associated hepatitis (Supplementary Fig. 1A, B)^4^. Treatment with prednisolone and mycophenolate led to clinical improvement but incomplete biochemical remission. He also exhibited toenail fragility and recurrent oral thrush without detectable IL-17A or IL-17F autoantibodies.

**Fig. 1:**
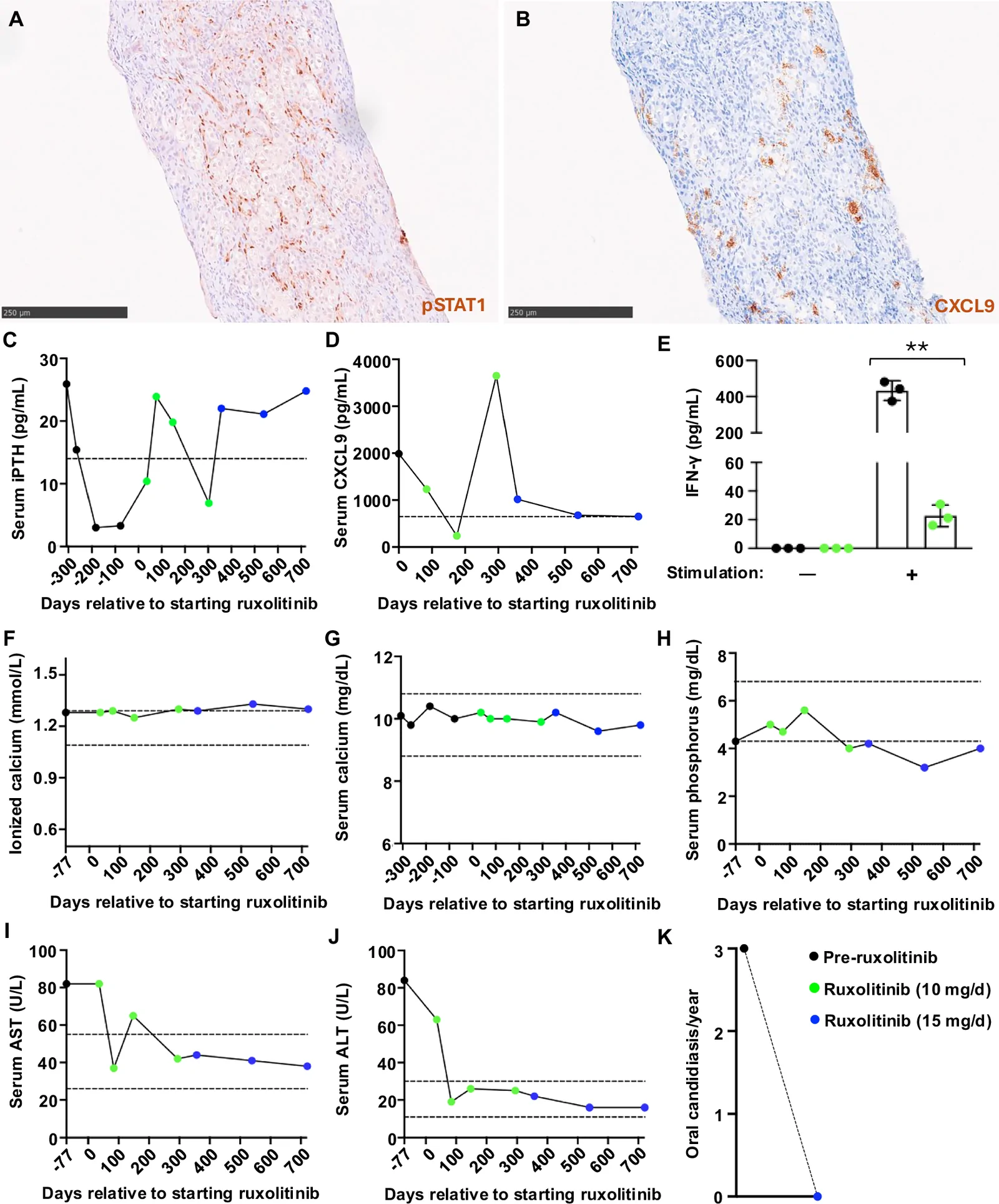
Ruxolitinib reverses evolving hypoparathyroidism and ameliorates refractory hepatitis and oral candidiasis in Patient 1. **A**, **B** Representative histological images (performed once) of liver biopsy at the time of autoimmune hepatitis diagnosis. **A** Immunostain for pSTAT1. **B** In situ hybridization for CXCL9. **C**–**K** Response to ruxolitinib in Patient 1. Data points in black represent timepoints before ruxolitinib initiation, whereas those in green and blue represent timepoints while receiving ruxolitinib at the indicated dose. **C** Serum iPTH over time relative to ruxolitinib initiation. The dashed horizontal line represents the lower limit of normal for this assay, and the dotted horizontal line represents the lower limit of detection (LOD) for this assay (<3 pg/mL). **D** Serum CXCL9 levels over time relative to ruxolitinib initiation. The dashed horizontal line marks the upper limit of normal for this assay. **E** T cell-derived IFN-γ production ex vivo post-CD3/CD28 stimulation (**P = 0.0051 by unpaired two-tailed t test with Welch’s correction). Each data point represents a technical replicate (*n* = 3 technical replicates for each condition). Error bars represent the mean +/− standard deviation. **F**–**H** Serum ionized calcium (**F**), total calcium (**G**), and phosphorus (**H**) levels over time relative to ruxolitinib initiation. **I**, **J** Serum AST and ALT levels over time relative to ruxolitinib initiation. The dashed horizontal lines in (**F**–**J**) represent the upper and lower limits of normal for the corresponding assays. **K** Episodes of oral candidiasis averaged over 1 year before and after ruxolitinib initiation.

Serial endocrine surveillance revealed a progressive decline in intact parathyroid hormone (iPTH), which decreased to near the lower limit of detection levels, fluctuating between undetectable (< 3 pg/mL) and low detectable values (~ 3.3 pg/mL) within four months. Despite this, calcium and phosphorus levels remained normal without requiring calcium or cholecalciferol replacement at that time, consistent with evolving subclinical autoimmune hypoparathyroidism. Serum CXCL9 (a chemokine induced by IFN-γ and a surrogate marker of pathway activity)^15^ was elevated (1988 pg/mL; normal, < 647 pg/mL) and liver tissue showed increased pSTAT1 and CXCL9 staining (Fig. 1A, B), whereas liver tissue from an APECED patient without autoimmune hepatitis lacked detectable staining for both markers (Supplementary Fig. 1C–F), indicating augmented IFN-γ signaling in blood and affected tissue.

Given that hypoparathyroidism affects ~ 85% of APECED patients and causes substantial morbidity and mortality^1,3^ and in light of the imminent risk of irreversible parathyroid failure, ruxolitinib was initiated at 5 mg twice daily (0.65 mg/kg/day) at age 28 months, with acyclovir prophylaxis to mitigate herpesvirus reactivation risk. At treatment initiation, the patient was receiving mycophenolate monotherapy, with prednisolone discontinued 80 days earlier. Within one month, iPTH increased to 10.4 pg/mL and reached 23.9 pg/mL by three months (Fig. 1C), while calcium and phosphorus levels remained normal. Serum CXCL9 declined to 240 pg/mL, and T cell-derived IFN-γ production decreased (Fig. 1D, E).

After eight months of treatment, iPTH declined in parallel with a 20% weight increase and a relative dose reduction to 0.55 mg/kg/day, accompanied by a concurrent rise in serum CXCL9 to 3651 pg/mL. Escalation of ruxolitinib to alternating 5-mg and 10-mg doses (0.79 mg/kg/day) restored and stabilized iPTH above 20 pg/mL within two months, with CXCL9 decreasing to 677 pg/mL. After two years of therapy, iPTH, calcium, and phosphorus levels remained normal (Fig. 1F–H) and the patient did not develop clinical hypocalcemia.

Beyond endocrine stabilization, toenail dystrophy resolved and AST/ALT normalized within three months of ruxolitinib, achieving complete biochemical remission of autoimmune hepatitis for the first time since diagnosis (Fig. 1I, J). Mycophenolate was discontinued five months after ruxolitinib initiation, and hepatic enzymes have remained normal on ruxolitinib monotherapy. No CMC recurrences occurred during two years of treatment, even during an antibiotic course for otitis media (Fig. 1K). Ruxolitinib-associated remission of CMC occurred without changes in IL-22 or type-I interferon autoantibody titers (Supplementary Fig. 2). Treatment was well-tolerated, without evidence of myelosuppression or impaired linear growth (Supplementary Fig. 3). Transient asymptomatic EBV and BK viremia were detected by surveillance PCRs concurrent with the CXCL9 increase to 3651 pg/mL and resolved spontaneously without clinical sequelae. The patient had no detectable CMV or JC viremia throughout treatment.

### Patient 2

An 18-year-old woman with APECED (homozygous *AIRE* c.967_979del) with a history of CMC, hypoparathyroidism, adrenal insufficiency, and enamel hypoplasia developed clinical and biochemical features of evolving hypergonadotropic hypogonadism. Laboratory evaluation revealed declining anti-Müllerian hormone (AMH; nadir 0.45 ng/mL, normal 1.2–12 ng/mL), undetectable estradiol during the mid-cycle phase (nadir, < 25 pg/mL, normal 38–649 pg/mL mid-cycle), and markedly elevated gonadotropins in the follicular phase, including follicle-stimulating hormone (FSH; 91.4 IU/L, normal, 3.0–8.1 IU/L) and luteinizing hormone (LH; 66.4 IU/L, normal 1.8-11.8 IU/L). She reported severe hot flashes and oligomenorrhea, with three menses over five months compared with previously regular 28-day cycles since menarche at age 13. Serum testing revealed autoantibodies against the steroidogenic side-chain cleavage (SCC) enzyme, which has been associated with autoimmune hypogonadism in APECED^16^.

**Fig. 2:**
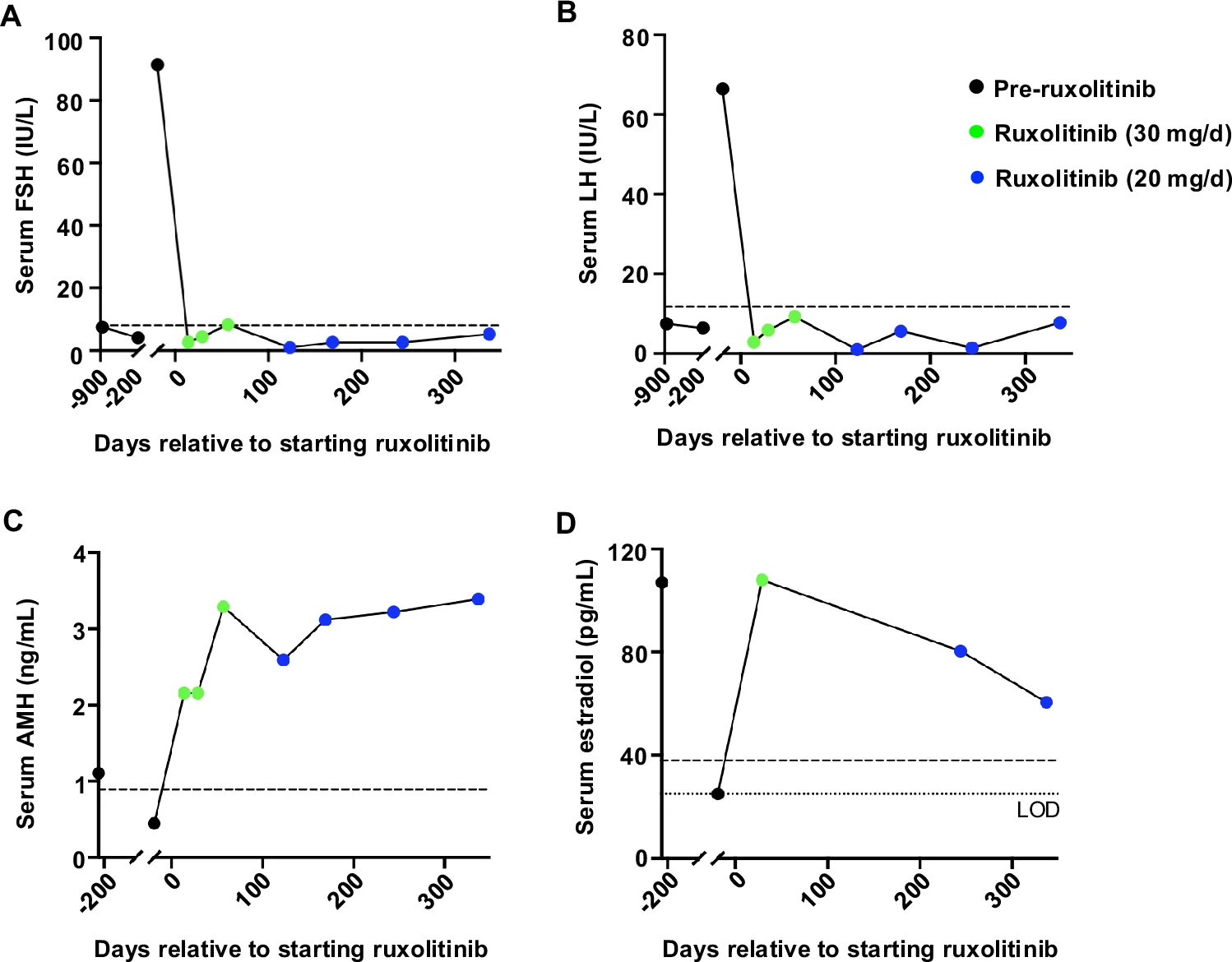
Ruxolitinib reverses premature ovarian failure in Patient 2. Data points in black represent timepoints before ruxolitinib initiation, whereas those in green and blue represent timepoints while receiving ruxolitinib at the indicated dose. **A**, **B** Serum FSH and LH levels over time relative to ruxolitinib initiation. **C** Serum AMH levels over time relative to ruxolitinib initiation. **D** Serum estradiol levels drawn in the mid-cycle phase over time relative to ruxolitinib initiation. The dashed lines represent the upper (**A**, **B**) or the lower (**C**, **D**) limits of normal for these assays [in the follicular phase for (**A**, **B**) or the mid-cycle for (**D**)]. The dotted line in (**D**) represents the lower limit of detection (LOD) (< 25 pg/mL).

Given the risk of irreversible ovarian insufficiency, ruxolitinib was initiated at 15 mg twice daily (0.59 mg/kg/day) with valacyclovir prophylaxis. Hot flashes diminished within one week and resolved by two weeks. Concurrently, FSH, LH, estradiol, and AMH normalized and remained stable over eleven months of therapy (Fig. 2A–D). Regular 28-day menstrual cycles resumed within three months. Ruxolitinib caused no hematopoietic, hepatic, or renal toxicity. A 6.8 kg weight gain prompted dose reduction to 10 mg twice daily (0.34 mg/kg/day) at three months, after which weight normalized without recurrence of clinical and biochemical hypogonadism.

## Discussion

These observations provide the first clinical evidence that targeted JAK1/2 inhibition can intercept evolving autoimmune endocrinopathies in APECED before irreversible endocrine glandular failure occurs. Ruxolitinib stabilized and reversed hypoparathyroidism and hypergonadotropic hypogonadism at an early stage of disease, conditions historically regarded as irreversible once established. These findings support the concept that, although autoimmune endocrine destruction is typically progressive, a therapeutic window may exist early in disease during which glandular function can be preserved with targeted immunomodulation^17,18^. Together, these data reposition autoimmune endocrine failure in APECED from an irreversible outcome to a modifiable, time-sensitive immunologic process. Beyond its endocrine benefits, ruxolitinib also induced remission of treatment-refractory autoimmune hepatitis and oral candidiasis. Notably, remission of CMC despite negative anti-IL-17A/IL-17F autoantibodies and unchanged anti-IL-22 titers supports an IFN-γ–dominant, T cell-driven, antibody-independent mechanism, consistent with our prior work^7,8^. This highlights a unified therapeutic effect across the fungal, endocrine, and non-endocrine manifestations of APECED, driven by excess IFN-γ signaling^7,8^.

Notably, a JAK1-selective inhibitor (upadacitinib) has also been reported to induce multiorgan remission in APECED^11^, suggesting that more selective JAK inhibition may be effective. However, because JAK inhibitors are relatively rather than absolutely selective, further studies will be required to define the comparative efficacy and safety of selective JAK1 or JAK2 inhibition relative to ruxolitinib, particularly in pediatric populations^12^.

Both patients tolerated ruxolitinib well, including a two-year-old child, supporting the feasibility of JAK1/2 inhibition in pediatric APECED. Dose reduction in the older patient maintained endocrine remission, suggesting that durable disease control may be achievable with lower maintenance dosing to minimize long-term toxicity associated with JAK inhibition, including weight gain, increased risk of infection, anemia, thrombosis, and non-melanoma skin cancers.

Patient 2 developed dose-dependent weight gain that resolved with dose reduction. This may relate to inhibition of JAK2-dependent leptin receptor signaling^19,20^. Although improved enteritis and nutrient absorption may contribute to weight gain in some APECED patients treated with ruxolitinib^8,9^, neither patient had clinical evidence of malabsorption.

Patient 1 experienced transient asymptomatic BK and EBV viremia. These events occurred during a period of elevated CXCL9, consistent with relative underdosing of ruxolitinib. Given the patient’s young age, it is unclear whether this reflected primary infection or viral reactivation; however, resolution of viremia despite increasing the ruxolitinib dose argues against sustained immunosuppression as the primary driver. To enable early detection of potential viral reactivation, we perform structured surveillance for BK, JC, CMV, and EBV viremia at least every 6 months.

Given the increased susceptibility of patients with APECED to severe herpesvirus infections^21^ and the potential for JAK inhibition to further increase this risk^22^, both patients received HSV/VZV prophylaxis with acyclovir or valacyclovir. Neither developed clinical HSV or VZV disease.

In Patient 1, serum CXCL9 levels generally paralleled IFN-γ pathway activity and therapeutic response, supporting its potential utility as a pharmacodynamic biomarker of ruxolitinib activity in APECED. However, these observations remain exploratory and will require validation in larger cohorts. These findings are consistent with prior studies in IFN-γ-driven conditions such as hemophagocytic lymphohistiocytosis^23^ and macrophage activation syndrome^24^ where CXCL9 reflects disease activity.

Long-term follow-up and treatment of a larger number of patients will be essential to determine the generalizability and durability of these responses, define optimal weight-based daily ruxolitinib dosing for prevention of endocrinopathies, and establish the long-term safety profile of JAK1/2 inhibition in APECED. Patient 1 illustrates the importance of close weight monitoring and weight-based dose adjustment in growing children receiving ruxolitinib, as normal growth can reduce mg/kg drug exposure over time. Longer-term follow-up of Patient 2, including gynecological surveillance, will be required to determine the durability of these responses and assess fertility outcomes.

This study is limited by the small number of treated patients. However, the depth of longitudinal biochemical, clinical, and biomarker follow-up provides mechanistic insight and translational proof-of-concept for endocrine disease interception. These observations support prospective, endocrine-focused clinical trials evaluating IFN-γ or JAK inhibition as tissue-preserving therapies in APECED. For Patient 2, the lack of direct ovarian tissue data limits mechanistic attribution. The presence of SCC autoantibodies supports autoimmune ovarian involvement^16^, but direct evidence of IFN-γ–mediated ovarian injury in humans with APECED remains lacking. Although increased IFN-γ signaling has been demonstrated in ovarian tissue in Aire-deficient mice^8^, the mechanistic link in humans remains inferential.

Collectively, these observations advance a disease-interception paradigm in which early recognition of APECED enables timely JAK1/2 inhibition to prevent progression to organ-specific autoimmune destruction, underscoring the value of early molecular diagnosis and structured endocrine surveillance in affected individuals^1^. Conceptually, this is a genotype-first precision-medicine strategy that links defined *AIRE* lesions to their pathogenic cytokine circuits to guide targeted therapy. Although demonstrated here in a monogenic disorder of failed central tolerance, these findings raise the possibility that analogous IFN-γ-driven mechanisms may contribute to autoimmune pathology in selected monogenic or polygenic immune-mediated conditions, including trisomy 21^25^, thymoma-associated autoimmunity^5^, and APS-2/APS-3^26^. Given that nonsurgical hypoparathyroidism affects 1.6-9.25 per 100,000 people^27^ (including rare cases in systemic lupus erythematosus^28,29^), premature ovarian insufficiency occurs in approximately 1000 per 100,000 women (with an estimated autoimmune contribution of up to 55%)^30^, and Hashimoto’s thyroiditis, an autoimmune disorder in which IFN-γ has been implicated^31^, affects approximately 7.5% of the global population^32^, these observations motivate further mechanistic and clinical investigation into whether pathway-informed targeted immunomodulation could preserve endocrine function in broader autoimmune contexts.

## Methods

### Study participants

Patients were admitted to the National Institutes of Health (NIH) Clinical Center and enrolled onto an IRB-approved prospective natural history research study (clinicaltrials.gov, NCT01386437) under which they underwent systematic clinical evaluation as previously described^3^. Informed consent was obtained from the parents of both patients’ at initial enrollment, with assent from Patient 2, who was a minor at the time, and subsequently provided informed consent upon reaching 18 years of age. Consent for publication of the information presented in this manuscript was obtained from Patient 1’s parents and from Patient 2. Both patients were managed and monitored by the NIH (J.P., T.W., and M.S.L.) and their local providers (H.G., M.S., S.M., A.B., K.L., and N.R. for Patient 1; N.M. for Patient 2). No commercial entity played a role in the study design or execution.

### Clinical laboratory testing

All clinical laboratory measurements were performed in CLIA-certified clinical laboratories. Serum iPTH levels were measured using an electrochemiluminescence immunoassay. Serum CXCL9 levels were measured using a validated microfluidics immunoassay.

### T cell activation culture using CD3/CD28 Dynabeads

Frozen PBMCs were harvested from Patient 1 before and at 6 months after treatment initiation with ruxolitinib and frozen. Following thawing at 37 °C in a water bath, the cells were washed once and adjusted to 1 × 10^6^ cells/ml in complete media (RPMI1640 + 10% fetal bovine serum + 100 ug/ml penicillin/streptomycin). Then, 2 × 10^5^ PBMCs were transferred to 96-well U bottom plates and left to acclimate for 1 h in a tissue culture incubator (37 °C, 5% CO2, 90–95% relative humidity). PBMCs were then stimulated for 72 h with Dynabeads Human T-Activator CD3/CD28 beads (Gibco, USA. Cat#11161D) per the manufacturer’s instructions. The plate was spun down (300 x *g*, 10 min, 4 °C), and IFN-γ was measured in the supernatant using a commercial ELISA kit (R&D Systems, USA. Cat#DY285B-05). Each experimental condition had three technical replicates.

### Autoantibody detection

Plasma from Patient 1 was screened for autoantibodies against interferon-α (IFN-α), IFN-β, IFN-γ, IFN-ω, GM-CSF, IL-12, IL-17A, IL-17F, IL-22, and IL-23 both before and after 1 year of ruxolitinib therapy using a multiplex particle-based assay as previously described^3^. In brief, differentially fluorescing magnetic beads were covalently coupled to recombinant human proteins (2.5 μg per reaction). Patient plasma was incubated for 30 minutes with combined beads (1:10,000 dilution). Beads were then washed and further incubated with PE-labeled goat anti-human IgG (1 μg/mL, eBioscience) for an additional 30 min. After an additional wash, beads were resuspended in assay buffer and analyzed on a Bio-Plex X200 instrument (Bio-Rad). Plasma from Patient 2 was screened for autoantibodies to the steroidogenic SCC enzyme using Luciferase immunoprecipitation systems (LIPS), as previously described^3,33^.

### Immunohistochemical evaluation of formalin-fixed paraffin-embedded liver tissue for CD4, CD8, and pSTAT1

Slides were baked for 60 min before staining. CD4/CD8 double immunohistochemical staining was performed on an automated immunostainer, BenchMark Ultra (Roche). After baking, Ultra CC1 (Roche, Cat No. 950-224) was applied to the slide for a 64 min antigen retrieval. Predilute CD4 (Clone SP35, Roche, Cat No. 790-4423) was detected first with a 40 min antibody incubation and detection with an ultraView Universal DAB Detection Kit (Roche, Cat No. 760-500). Predilute CD8 (Clone SP57, Roche, Cat No. 790-4460) was then applied for a 32 min incubation followed by detection with an ultraView Universal Alkaline Phosphatase Red Detection Kit (Cat No. 760-501). Hematoxylin was used as a counterstain. The slides were dehydrated in graded alcohols, treated with xylene (2 × 5 min), and cover-slipped.

pSTAT1 (Tyr701) immunohistochemical staining was performed using the BenchMark Ultra automated immunostainer. Antigen retrieval was performed using Ultra CC1 for 64 min. A 1:200 dilution of pSTAT1 Rabbit mAb (Clone 58D6, Cell Signaling Technology, Cat No. 9167) in Dako antibody diluent (Agilent Dako, Cat No. S0809) was applied and incubated for 60 min followed by detection with an ultraView Universal DAB Detection Kit with counterstaining with Hematoxylin. The slides were dehydrated in graded alcohol, treated with xylene (2 × 5 min), and cover-slipped.

### In situ hybridization-based detection of CXCL9 in liver tissue

Slides were baked for 60 min prior to staining. As previously described^8^, the manual RNAscope® 2.5 High Definition (HD)- BROWN Assay kit (Advanced Cell Diagnostics, Inc. (ACDBio), Cat No. 322300) was used according to the manufacturer’s instructions to perform in situ hybridization. Slides with formalin-fixed, paraffin-embedded tissue were deparaffinized in xylene (2  × 5 min), dehydrated in 100% EtOH (2 × 1 min), air dried at room temperature, and incubated in RNAscope® Hydrogen Peroxide (Cat No. 322330, ACDBio) at room temperature for 10 min to quench endogenous peroxidases. Slides were washed in DI water twice before being submerged in 700 mL of fresh boiling RNAscope® 1X Target Retrieval Reagents solution (Cat No. 322000, ACDBio) for 15 min and then washed twice in DI water, once in 100% EtOH, and air dried at room temperature. A hydrophobic barrier was drawn around the tissue, and RNAscope® Protease Plus (Cat No. 322330, ACDBio) was applied for 20 min in a HybEZ™ Oven at 40 °C (ACDBio). Then, slides were washed twice in DI water and incubated with Hs-CXCL9 Probe (Cat No. 440161) for 2 h in the HybEZ™ Oven. RNAscope® signal amplification reagents (Cat No. 322310, ACDBio) AMP 1 (30 min), AMP 2 (15 min), AMP 3 (30 min), AMP 4 (15 min), AMP 5 (75 min), and AMP 6 (15 min) were then applied and incubated in the HybEZ™ Oven. The slides were washed twice with RNAscope® 1X Wash Buffer (Cat No. 310091, ACDBio) before each AMP reagent was added. After removing AMP 6, slides were washed twice in wash buffer, and RNAscope® DAB detection reagents (Cat No. 322310, ACDBio) were applied, and slides were incubated for 10 min in the HybEZ™ Oven. Sections were washed in tap water, counterstained with HarrisMayers Hematoxylin, washed again in tap water, placed in 0.02% ammonia water for 10 s, and washed with tap water. Sections were then dehydrated in graded alcohols, treated with xylene (2  × 5 min), and cover-slipped.

### Statistical analyses

Statistical analyses and graphical visualizations were carried out in GraphPad Prism version 10.2.0 (GraphPad Software LLC). An unpaired two-tailed t test with Welch’s correction was used for T cell stimulation-induced IFN-γ level comparisons (Fig. 1E; *n* = 3 technical replicates for each condition).

### Reporting summary

Further information on research design is available in the Nature Portfolio Reporting Summary linked to this article.

## Supplementary information


Supplementary Information
Transparent Peer Review file
Reporting Summary


## Source data


Source Data


## Data Availability

The data supporting the findings of the study are available within the article and its Supplementary Information files. Source data are provided in this paper.
