# Supplementary material for: Ruxolitinib prevents irreversible autoimmune endocrinopathies in APECED
Source: Nat Commun. 2026 Aug 1;17:9295. doi: 10.1038/s41467-026-76285-x (PMC13526882; doi:10.1038/s41467-026-76285-x)
Supplement: Supplementary file 1 [file 41467_2026_76285_MOESM1_ESM.pdf]

Ruxolitinib prevents irreversible autoimmune endocrinopathies in APECED

Joseph Pechacek[1], Taura Webb[1], Lucas dos Santos Dias[1], Rachel Wu[2], Heather Moorman[1], Princess Barber[1], Lindsey B. Rosen[3], Peter D. Burbelo[4], Benjamin Colton[5], Herodes Guzman[6,7], Maria Sacta[8], Shana E. McCormack[6], Aaron Bennett[9], Kathleen Loomes[9], Neil Romberg[8], Niamh McGrath[10], Steven M. Holland[3], Theo Heller[11], Karen K. Winer[12], Stefania Pittaluga[2], Michail S. Lionakis[1]*

[1]Fungal Pathogenesis Section, Laboratory of Clinical Immunology and Microbiology (LCIM), National Institute of Allergy and Infectious Diseases (NIAID), National Institutes of Health (NIH), Bethesda, MD, USA
[2]Laboratory of Pathology, National Cancer Institute, NIH, Bethesda, MD, USA
[3]Immunopathogenesis Section, LCIM, NIAID, NIH, Bethesda, MD, USA
[4]Adeno-Associated Virus Biology Section, National Institute of Dental and Craniofacial Research (NIDCR), NIH, Bethesda, Maryland, USA.
[5]Pharmacy Department, NIH Clinical Center, Bethesda, Maryland, USA.
[6]Division of Endocrinology and Diabetes, Children's Hospital of Philadelphia, Philadelphia, PA, USA
[7]Division of Human Genetics, Children's Hospital of Philadelphia, Philadelphia, PA, USA
[8]Division of Allergy and Immunology, Children's Hospital of Philadelphia, Philadelphia, PA, USA
[9]Division of Gastroenterology, Hepatology, and Nutrition, Children's Hospital of Philadelphia, Philadelphia, PA, USA
[10]School of Public Health, University College Dublin, Dublin, Ireland
[11]Liver Diseases Branch, National Institute of Diabetes and Digestive and Kidney Diseases, NIH, Bethesda, MD, USA
[12]Pediatric Growth and Nutrition Branch, National Institute of Child Health and Human Development, NIH, Bethesda, MD, USA
*Correspondence: lionakism@mail.nih.gov

Table of contents:

Figure S1: Histological evaluation of liver tissue in Patient 1 and a control APECED patient without autoimmune hepatitis — Page 2

Figure S2: Anti-cytokine autoantibodies in Patient 1 over time on ruxolitinib — Page 3

Figure S3: Safety of ruxolitinib in Patient 1 over two years of therapy — Page 4

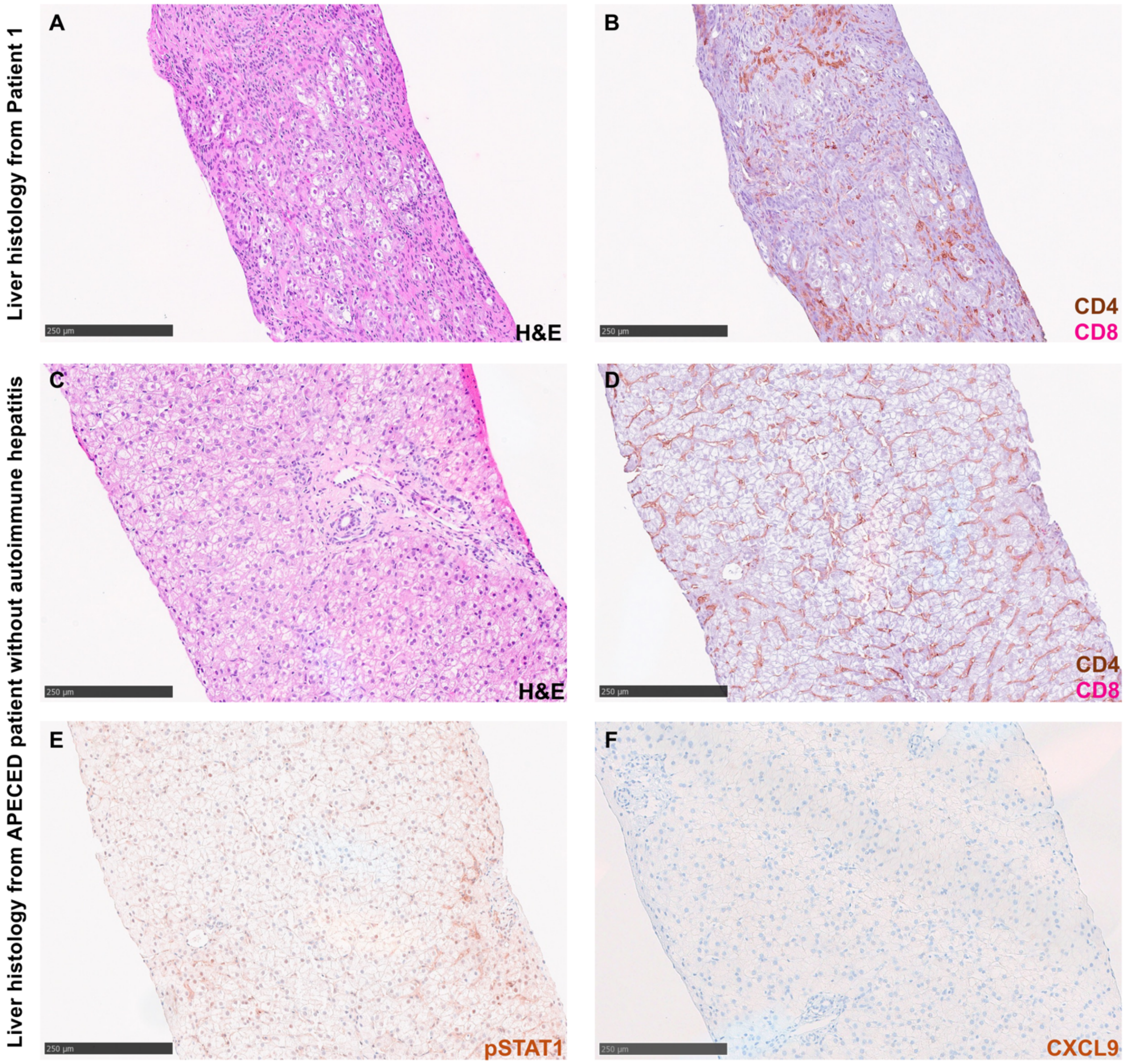

Supplementary Figure 1. Histological evaluation of liver tissue in Patient 1 and a control APECED patient without autoimmune hepatitis. A-B) Shown are representative (A) hematoxylin and eosin (H&E) and (B) CD4 (brown) and CD8 (red) immunostaining obtained from biopsy of Patient 1's liver at the time of diagnosis with autoimmune hepatitis. C-F) Shown are representative liver images from a control APECED patient with histological evidence of diffuse glycogenosis but no autoimmune hepatitis, including (C) H&E, (D), CD4 (brown) and CD8 (red) immunostaining, (E) pSTAT1 immunostaining, and (F) CXCL9 in situ hybridization. Note CD4 faint uptake by Kupffer cells lining hepatic sinusoids in panels B and D and background non-specific staining of pSTAT1 in panel E, which contrasts the dark nuclear staining seen in the liver of Patient 1 with autoimmune hepatitis in Figure 1A.

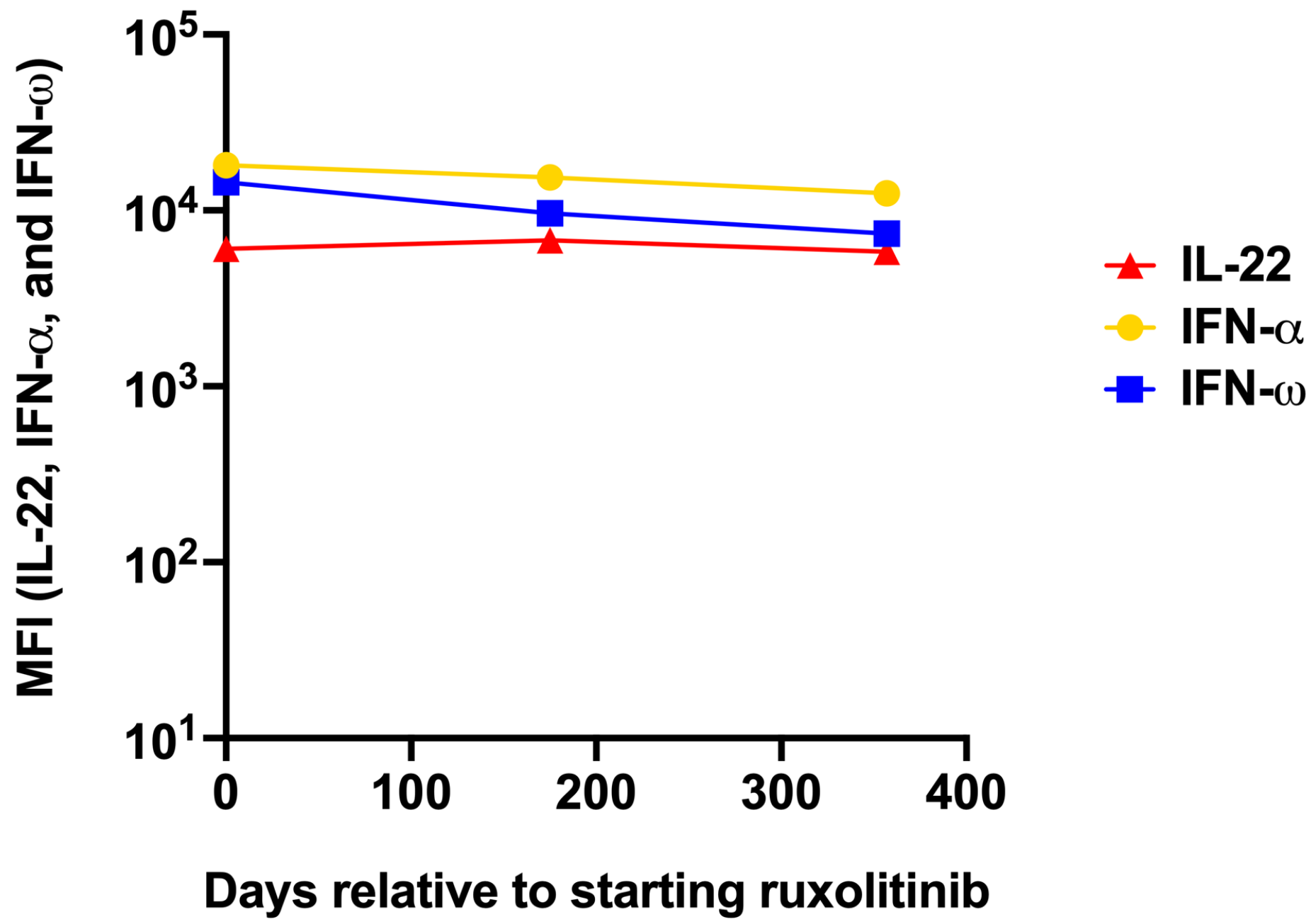

Supplementary Figure 2. Anti-cytokine autoantibodies in Patient 1 over time on ruxolitinib. Titers of IL-22, interferon-α (IFN-α) and interferon-ω (IFN-ω) expressed as mean fluorescence intensity (MFI) using a particle-based assay in Patient 1's plasma. The three depicted MFI values from left to right correspond to timepoints before ruxolitinib initiation, while receiving ruxolitinib at 5 mg twice daily, and while receiving ruxolitinib at alternating doses of 5 mg and 10 mg, respectively. Autoantibodies to interferon-β, interferon-γ, GM-CSF, IL-12, IL-17A, IL-17F, and IL-23 were found to be negative at all three measured timepoints (not depicted).

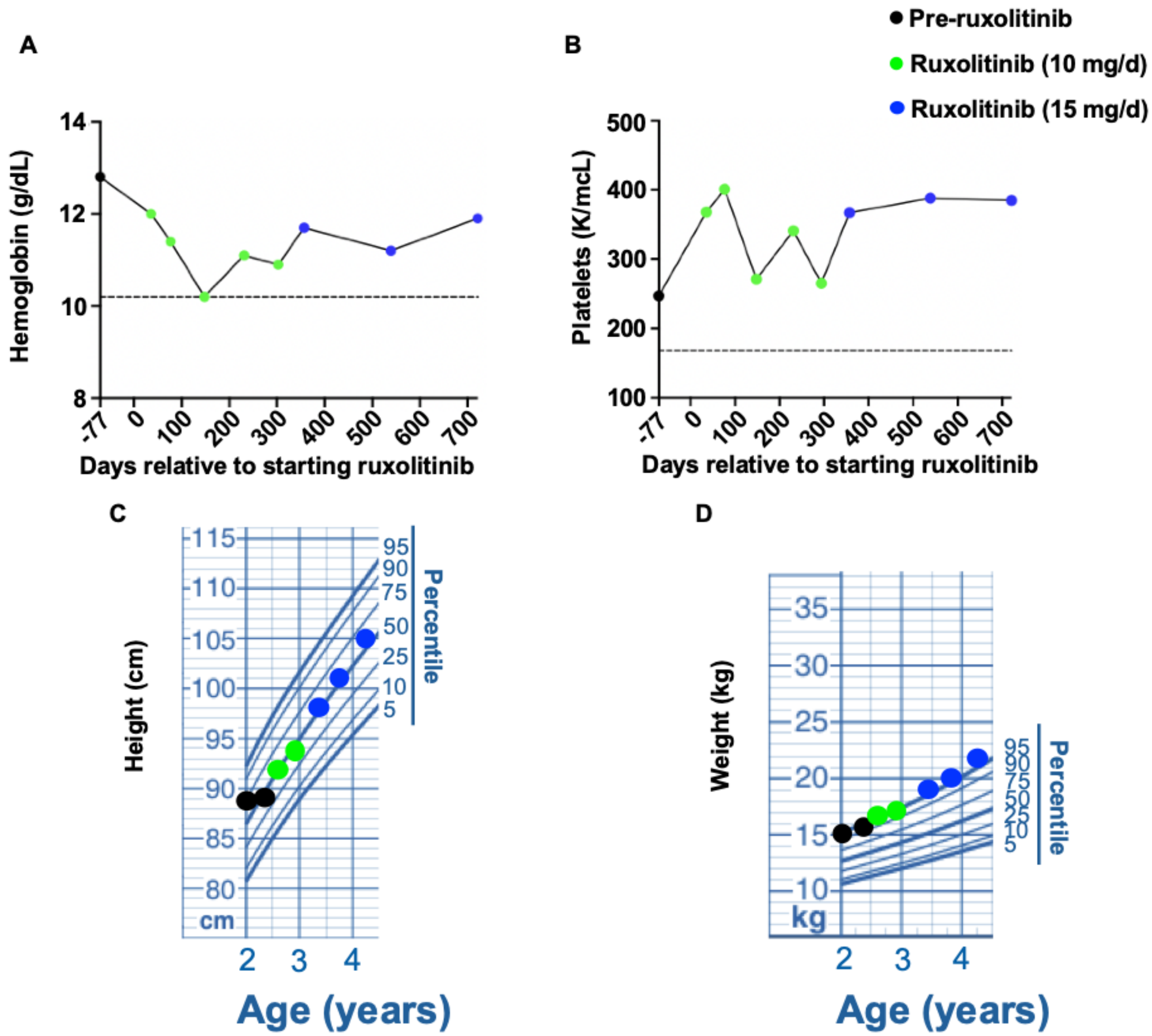

Supplementary Figure 3. Safety of ruxolitinib in Patient 1 over two years of therapy. A-B) Data points in black represent timepoints before ruxolitinib initiation whereas those in green and blue represent timepoints while receiving ruxolitinib at the indicated dose. A) Hemoglobin levels over time relative to ruxolitinib initiation. B) Platelet counts over time relative to ruxolitinib initiation. The dashed horizontal line in A-B represents the lower limit of normal for these assays. C-D) Growth graphed on Center for Disease Control growth charts over time spanning the period receiving ruxolitinib. C) Linear growth over time. D) Weight over time.
