# Supplementary material for: Ruxolitinib prevents irreversible autoimmune endocrinopathies in APECED
Source: Nat Commun. 2026 Aug 1;17:9295. doi: 10.1038/s41467-026-76285-x (PMC13526882; doi:10.1038/s41467-026-76285-x)
Supplement: Supplementary file 2 [file 41467_2026_76285_MOESM2_ESM.pdf]

nature portfolio

Peer Review File

Ruxolitinib prevents irreversible autoimmune endocrinopathies in APECED

Corresponding Author: Dr Michail Lionakis

This file contains all reviewer reports in order by version, followed by all author rebuttals in order by version.

Version 0:

Reviewer comments:

Reviewer #1

(Remarks to the Author)
This is an important pair of case reports, challenging the prevailing paradigm that APECED-associated autoimmune endocrinopathy is inevitably irreversible, and instead highlighting the valuable potential to prevent lifelong dependence on hormonal supplementation. The authors have already contributed the extremely valuable insight that APECED-associated disease is heavily interferon-gamma-dependent and shown remarkable benefit from JAK inhibition in ameliorating a variety of APECED manifestations and associated disease biomarkers. Here they extend this principle to young people with biochemical features of incipient endocrine failure (parathyroid and ovarian, respectively) and demonstrate the longed for ability to turn back the tide of autoimmunity.

The clinical and laboratory data tell a simple but compelling story. If confirmed, this effect implies the need for a fundamental change in the management of APECED – the opportunity to prevent end organ damage including endocrinopathy – as well as a re-evaluation of its potential relevance to a wider range of autoimmune conditions. Of course the longevity of such an effect, its generalizability and the safety of longterm JAK inhibition remain to be established, as the authors point out. It is conceivable that benefits will be shortlived upon release from JAK inhibition (similar to the situation with CD3 modulators in T1D) or even that the immune system may divert to an alternative pathological pathway in the face of ongoing blockade. But this clinician, and no doubt the APECED patient community at large, will be cheering these authors along with their plan for a controlled trial to establish how best to use these new tools.

My suggestions for revision are confined to minor syntactical/typographic details:

Para 2, page 3: "We recently showed that excessive interferon- (IFN-) is a dominant driver of autoimmune tissue injury and CMC in APECED6,7 and that inhibition of Janus kinases 1/2 (JAK1/2) with ruxolitinib ameliorates inflammatory and infectious manifestations in both Aire-deficient mice and patients with APECED has shown efficacy in ameliorating CMC and several organ-specific autoimmune manifestations in APECED7-10." – this statement seems to represent the collision of 2 sentences that need splitting up again.

Para 2, page 4: "After eight months of treatment, iPTH declied" - presumably should read "…declined". Incidentally, I could not find a description of the method by which iPTH was measured – this seems particularly important given that this was the only indicator of incipient hypoparathyroidism in patient 1.

Penultimate para, page 5 "disease-preserving therapies in APECED." – do the authors mean to say either "disease-reversing" or perhaps "tissue-preserving"?

In figure 2 A-D, I can see that the sudden onset of incipient gonadal failure was what triggered the use of JAKi. Given the uncertainty about the evolution of this process, I'm uncomfortable with the joining of data points just before D-200 with those just before D0 & suggest a break in the x-axis should be placed here in every plot.

Reviewer #2

(Remarks to the Author)

Summary
The authors describe two individuals with APECED who developed early autoimmune hypoparathyroidism and hypergonadotropic hypogonadism. Treatment with the JAK1/2 inhibitor ruxolitinib halted progression and reversed biochemical and clinical abnormalities, These findings provide, to their knowledge, the first clinical evidence that timely JAK-STAT pathway inhibition can intercept endocrine autoimmunity in APECED.

General comment
These 2 cases describe similar results as the 3 cases reported in J Clin Immunol (Levy et al). Nevertheless, they show the complete regression of hypoparathyroidism when treated at the beginning of the disease, which was not the case in Levy's paper. The case about gonadal failure is more debatable since ovarian failure is sometimes fluctuating at onset. But the patient remained stable over one year
In addition, the manuscript provides physiopathological data on the immune mechanism and general thoughts about a new strategy of treatment to be investigated in autoimmunity.

Specific comments
Introduction
The authors should provide explanations about the pathway CXCL9 and STAT1.

Results
- PTH undetectable and normal calcium: how do the authors explain the maintenance of normal calcium without detectable PTH? Did the patients receive vitamin D supplementation? I have already observed this biological profile at the beginning of hypoparathyroidism.

- liver tissue showed increased pSTAT1 and CXCL9 staining. It would be interesting to show a liver control

- In both patients, weight gain was observed: what is the mechanism? a direct side effect of Ruxolitinib? an improvement of malabsorption? did the patients had diarrhea? steatorrhea? celiac disease or other autoimmune types of malabsorption as it has been reported in APECED? Did you investigate these aspects before and after treatment with ruxolitinib?

Discussion
- L 191: Our findings challenge the prevailing paradigm that autoimmune endocrine destruction is inexorably progressive: Indeed, as for type 1 diabetes, the destruction is progressive and acting at onset of the disease may be beneficial. For instance, the paper doi: 10.1210/jc.2005-1538 had already shown a regression of autoimmune manifestations with immunosuppression after kidney transplantation in a child: Autoimmune polyendocrinopathy-candidiasis-ectodermal dystrophy syndrome with renal failure: impact of posttransplant immunosuppression on disease activity. Otherwise, reversal of some autoimmune diseases is not uncommon in my experience in PEAI2 after islet transplantation in T1D especially for celiac disease, corticotrope insufficiency, Biermer disease and thyroiditis. At the opposite, resurgence of autoimmune disease after immunosuppression discontinuation has also been reported (doi: 10.2337/dc08-2339). So the sentence should be modified.

- L 201 : long-term toxicity: these long-term toxicities should be detailed

- serum CXCL9 levels closely paralleled IFN- activity: is CXCL19 easier to measure than IFN g

Formal comments
iPTH declied : replace by iPTH declined

Reviewer #3

(Remarks to the Author)
This paper describes 2 pediatric/young adult cases of APECED in whom ruxolitinib was started early, leading to stabilization and partial reversal of evolving hypoparathyroidism (Patient 1) and hypergonadotropic hypogonadism (Patient 2), along with remission of refractory autoimmune hepatitis and CMC. The proposed mechanism (IFN-gamma-driven endocrine glandular destruction intercepted by JAK1/2 inhibition) is scientifically sound and builds credibly on the authors' prior work. The longitudinal biochemical data are detailed and the clinical observations carry genuine significance. That said, several major concerns need to be addressed before this manuscript is ready for publication.
Major Comments
1. The claim: “first documented reversal of autoimmune hypoparathyroidism” needs to be qualified. Both Oikonomou et al. (NEJM 2024) and Lévy et al. (J Clin Immunol 2024; DOI: 10.1007/s10875-023-01629-x) have already reported improvements in PTH levels and calcium supplementation requirements under ruxolitinib in APS-1. The novelty here is more specifically the reversal of evolving subclinical hypoparathyroidism before irreversible glandular destruction has occurred. The authors should revise the claim accordingly and also clarify whether Patient 1 had any detectable residual parathyroid function at treatment initiation (e.g., measurable iPTH), as this is the biological basis for the “therapeutic window” concept they propose.
2. Patient 1 achieved CMC remission in the absence of detectable anti-IL-17A/F antibodies and without any change in anti-IL-22 titers during treatment (Extended Data Figure 2). This is mechanistically important, given that the canonical CMC pathogenesis in APECED involves neutralizing autoantibodies against IL-17A/F and IL-22. The persistence of remission

despite unchanged antibody titers is currently unexplained and deserves an explicit discussion. The authors should address whether this might represent an IFN-gamma-predominant, antibody-independent disease mechanism and what implications this has for the therapeutic rationale.
3. The extrapolation of the IFN-gamma paradigm to hypergonadotropic hypogonadism in Patient 2 is clinically compelling but lacks direct mechanistic grounding in the manuscript. While elevated pro-inflammatory cytokines including IFN-gamma have been reported in autoimmune premature ovarian insufficiency, direct evidence of IFN-gamma-mediated follicular destruction in APECED is not available. The authors should acknowledge this gap explicitly. They should also note the absence of steroid cell autoantibodies, and ovarian biopsy data, which would have strengthened mechanistic attribution. Fertility outcomes and the need for long-term gynecological surveillance are not mentioned and should be addressed.
4. Transient asymptomatic EBV and BK viremia were detected in Patient 1 during a period of relative under-dosing (low CXCL9). The authors attribute this to immune reconstitution rather than immunosuppression, but this interpretation is not argued explicitly. The choice of acyclovir prophylaxis , which is inactive against BK virus, also needs justification. A structured viral surveillance protocol should be described. Given that this is a young child receiving JAK1/2 inhibition, this safety signal warrants a dedicated paragraph in the Discussion rather than a brief mention.
5. The proposal to use serum CXCL9 to guide ruxolitinib dosing is internally consistent and clinically interesting. However, the normal reference threshold used is not accompanied by a cited source or validated assay platform. Moreover, no formal correlation between CXCL9 and iPTH values over time in Patient 1 is provided. The authors should either present a Pearson or Spearman correlation across available longitudinal time points, or explicitly label this a pilot/exploratory observation pending validation in a larger cohort. Contextualizing CXCL9 within the existing literature supporting it as an IFN-gamma-dependent disease activity marker (e.g. in macrophage activation syndrome) would also help anchor this claim.
Minor Comments
1. The claim that ruxolitinib achieves a “one-stop” multiorgan therapeutic effect would benefit from acknowledging that Hadjiyannis et al. (JACI 2025; DOI: 10.1016/j.jaci.2025.03.026) demonstrated comparable multiorgan remission with a selective JAK1 inhibitor. The Discussion should address whether JAK1 selectivity might offer a more favorable safety profile in the pediatric setting.
2. Weight gain as a potential pharmacokinetic confounder of mg/kg dosing is documented in Patient 1 but not discussed conceptually. The authors should recommend weight-based dose monitoring as a practical component of ruxolitinib management in growing children with APECED.
3. Typographical error Line 141: “iPTH declied” should read “declined.”

Version 1:

Reviewer comments:

Reviewer #1

(Remarks to the Author)
The authors have added additional commentary and information that accurately contextualizes their findings, although the modified discussion is now a little clunky and could be streamlined somewhat (eg discussion of JAKi-related weight-gain need not be revisited across multiple paragraphs).

As before I am enthusiastic about the importance of sharing these experimental medicine studies, which offer so much more than anecdote. By demonstrating modification of the underlying pathological process, this study highlights both the tools and the rationale for a pre-emptive strategy in the treatment of IFNg-driven autoimmunity.

Reviewer #2

(Remarks to the Author)
The authors have answered all my comments.

In my opinion, this manuscript is suitable for publication

Reviewer #3

(Remarks to the Author)
The manuscript was nicely revised according to suggestions given and it can be accepted for publications. Thanks.

Open Access This Peer Review File is licensed under a Creative Commons Attribution 4.0 International License, which permits use, sharing, adaptation, distribution and reproduction in any medium or format, as long as you give appropriate credit to the original author(s) and the source, provide a link to the Creative Commons license, and indicate if changes were made.
In cases where reviewers are anonymous, credit should be given to 'Anonymous Referee' and the source.
The images or other third party material in this Peer Review File are included in the article's Creative Commons license, unless indicated otherwise in a credit line to the material. If material is not included in the article's Creative Commons license and your intended use is not permitted by statutory regulation or exceeds the permitted use, you will need to obtain permission directly from the copyright holder.
To view a copy of this license, visit https://creativecommons.org/licenses/by/4.0/

RESPONSE TO REVIEWERS' COMMENTS

Reviewer 1:
We thank the reviewer for their positive and thoughtful assessment of our work, and for highlighting the potential clinical and conceptual implications of these findings.

Comment 1: *Para 2, page 3: "We recently showed that excessive interferon-γ (IFN-γ) is a dominant driver of autoimmune tissue injury and CMC in APECED[6,7] and that inhibition of Janus kinases 1/2 (JAK1/2) with ruxolitinib ameliorates inflammatory and infectious manifestations in both Aire-deficient mice and patients with APECED has shown efficacy in ameliorating CMC and several organ-specific autoimmune manifestations in APECED[7-10]." – this statement seems to represent the collision of 2 sentences that need splitting up again.*

Response: We thank the reviewer for noting this. The sentence has now been revised to remove redundant phrasing and improve clarity (Page 3, Lines 104-107).

Comment 2: *Para 2, page 4: "After eight months of treatment, iPTH declied" - presumably should read "…declined". Incidentally, I could not find a description of the method by which iPTH was measured – this seems particularly important given that this was the only indicator of incipient hypoparathyroidism in patient 1.*

Response: This typographical error has now been corrected (Page 4, Line 153). We have also added a description of the iPTH assay (electrochemiluminescence immunoassay) in the Methods of the revised manuscript (Page 7, Lines 297-301).

Comment 3: *Penultimate para, page 5 "disease-preserving therapies in APECED." – do the authors mean to say either "disease-reversing" or perhaps "tissue-preserving"?*

Response: We thank the reviewer for this suggestion. The term has been revised to "tissue-preserving" for clarity (Page 6, Line 260).

Comment 4: *In figure 2 A-D, I can see that the sudden onset of incipient gonadal failure was what triggered the use of JAKi. Given the uncertainty about the evolution of this process, I'm uncomfortable with the joining of data points just before D-200 with those just before D0 & suggest a break in the x-axis should be placed here in every plot.*
Response: We thank the reviewer for this helpful suggestion. We have introduced a break in the x-axis and removed the line connecting data points across this interval in the revised Figure 2A-D.

Reviewer 2:
We thank the reviewer for their positive and thoughtful assessment of our work, and for highlighting both the clinical implications of early intervention and the mechanistic insights underlying our findings.

Comment 1: *The authors should provide explanations about the pathway CXCL9 and STAT1.*

Response: We thank the reviewer for this suggestion. We have added a brief description of the IFN-γ-JAK-STAT1-CXCL9 pathway in the introduction to provide mechanistic context (Page 3, Lines 108-112).

Comment 2: *PTH undetectable and normal calcium: how do the authors explain the maintenance of normal calcium without detectable PTH? Did the patients receive vitamin D supplementation? I have already observed this biological profile at the beginning of hypoparathyroidism.*

Response: We thank the reviewer for this insightful comment. We agree that this biochemical profile is consistent with early/subclinical hypoparathyroidism. We have clarified in the revised manuscript that the patient was not receiving calcium or vitamin D supplementation at that time (Page 3, Line 134-137).

Comment 3: *Liver tissue showed increased pSTAT1 and CXCL9 staining. It would be interesting to show a liver control.*

Response: We thank the reviewer for this helpful suggestion. We have added representative control liver images from an APECED patient without autoimmune hepatitis stained for pSTAT1 and CXCL9, demonstrating lack of staining in the absence of autoimmune hepatitis (Supplementary Figure 1C-F). We have also incorporated this comparison into the revised Results section to further support the specificity of the observed IFN-γ signaling in affected liver tissue from Patient 1 (Page 4, Lines 141-142).

Comment 4: *In both patients, weight gain was observed: what is the mechanism? a direct side effect of Ruxolitinib? an improvement of malabsorption? did the patients had diarrhea? steatorrhea? celiac disease or other autoimmune types of malabsorption as it has been reported in APECED? Did you investigate these aspects before and after treatment with ruxolitinib?*

Response: We thank the reviewer for this important question. Neither patient had a history of diarrhea, steatorrhea, celiac disease, or other evidence of malabsorption. Patient 1's weight followed a stable growth percentile trajectory before and after ruxolitinib initiation (Figure S2). In contrast, Patient 2 developed dose-dependent weight gain that resolved with dose reduction, suggesting a pharmacologic effect, potentially related to inhibition of JAK2-mediated leptin receptor signaling. We have added a brief discussion and references related to these points in the revised manuscript (Page 5, Lines 225-229).

Comment 5: *L 191: Our findings challenge the prevailing paradigm that autoimmune endocrine destruction is inexorably progressive: Indeed, as for type 1 diabetes, the destruction is progressive and acting at onset of the disease may be beneficial. For instance, the paper doi: 10.1210/jc.2005-1538 had already shown a regression of*

autoimmune manifestations with immunosuppression after kidney transplantation in a child: Autoimmune polyendocrinopathy-candidiasis-ectodermal dystrophy syndrome with renal failure: impact of posttransplant immunosuppression on disease activity. Otherwise, reversal of some autoimmune diseases is not uncommon in my experience in PEAI2 after islet transplantation in T1D especially for celiac disease, corticotrope insufficiency, Biermer disease and thyroiditis. At the opposite, resurgence of autoimmune disease after immunosuppression discontinuation has also been reported (doi: 10.2337/dc08-2339). So the sentence should be modified.

Response: We agree with the reviewer. We have revised the sentence to better reflect the concept that autoimmune endocrine destruction is progressive, while emphasizing the potential for early intervention to preserve glandular function (Page 5, Lines 200-204). We have also added the suggested references to contextualize our findings within prior reports of autoimmune disease modulation with immunosuppression.

Comment 6: *Long-term toxicity: these long-term toxicities should be detailed.*

Response: We thank the reviewer for this important point. We have expanded the Discussion of the revised manuscript to detail long-term toxicities associated with JAK inhibition, including infection risk, hematologic effects, thrombosis, and non-melanoma skin cancers (Page 5, Lines 222-224).

Comment 7: *Serum CXCL9 levels closely paralleled IFN-γ activity: is CXCL9 easier to measure than IFNg.*

Response: We thank the reviewer for this important point. We agree that CXCL9 is more readily measurable than IFN-γ in serum and serves as a reliable surrogate marker of IFN-γ pathway activity. We have clarified this in the revised manuscript (Page 3, Line 138-139).

Comment 8: *iPTH declied: replace by iPTH declined*

Response: This typographical error has now been corrected (Page 4, Line 153).

Reviewer 3:
We thank the reviewer for their careful and thoughtful evaluation of our manuscript, and for recognizing the significance of the clinical observations and their mechanistic framework. We appreciate the insightful comments and suggestions provided below.

Major comment 1: *The claim: "first documented reversal of autoimmune hypoparathyroidism" needs to be qualified. Both Oikonomou et al. (NEJM 2024) and Lévy et al. (J Clin Immunol 2024; DOI: 10.1007/s10875-023-01629-x) have already reported improvements in PTH levels and calcium supplementation requirements under ruxolitinib in APS-1. The novelty here is more specifically the reversal of evolving subclinical hypoparathyroidism before irreversible glandular destruction has occurred. The authors should revise the claim accordingly and also clarify whether Patient 1 had*

any detectable residual parathyroid function at treatment initiation (e.g., measurable iPTH), as this is the biological basis for the "therapeutic window" concept they propose.

Response: We thank the reviewer for this important clarification. We have revised the manuscript to more precisely position our findings in the context of prior reports (2024 NEJM and J Clin Immunol). Specifically, we now emphasize reversal of evolving, early-stage hypoparathyroidism prior to irreversible glandular destruction, consistent with a therapeutic window for intervention (Page 5, Lines 204-206). We have also clarified that Patient 1 had residual parathyroid function prior to treatment initiation, with low but detectable iPTH levels (Page 3, Lines 134-137; Figure 1C).

Major comment 2: *Patient 1 achieved CMC remission in the absence of detectable anti-IL-17A/F antibodies and without any change in anti-IL-22 titers during treatment (Extended Data Figure 2). This is mechanistically important, given that the canonical CMC pathogenesis in APECED involves neutralizing autoantibodies against IL-17A/F and IL-22. The persistence of remission despite unchanged antibody titers is currently unexplained and deserves an explicit discussion. The authors should address whether this might represent an IFN-gamma-predominant, antibody-independent disease mechanism and what implications this has for the therapeutic rationale.*

Response: We thank the reviewer for this important point. We have expanded the Discussion to note that remission of CMC occurred despite negative anti-IL-17A/IL-17F autoantibodies and unchanged anti-IL-22 titers, supporting an IFN-γ–dominant, T cell-driven, antibody-independent mechanism, consistent with our prior work (PMID: 33446526 and 38810185). We also clarify the implications of this observation for the therapeutic rationale of targeting the IFN-γ-JAK-STAT pathway in APECED (Page 5, Lines 208-212).

Major Comment 3: *The extrapolation of the IFN-gamma paradigm to hypergonadotropic hypogonadism in Patient 2 is clinically compelling but lacks direct mechanistic grounding in the manuscript. While elevated pro-inflammatory cytokines including IFN-gamma have been reported in autoimmune premature ovarian insufficiency, direct evidence of IFN-gamma-mediated follicular destruction in APECED is not available. The authors should acknowledge this gap explicitly. They should also note the absence of steroid cell autoantibodies, and ovarian biopsy data, which would have strengthened mechanistic attribution. Fertility outcomes and the need for long-term gynecological surveillance are not mentioned and should be addressed.*

Response: We thank the reviewer for this important point. We have tempered the mechanistic interpretation and now explicitly acknowledge the absence of direct ovarian tissue data in this patient (Page 6, Lines 260-265). We additionally report the presence of steroidogenic side-chain cleavage (SCC) autoantibodies, supporting autoimmune ovarian involvement in this case (Pages 5-6, Lines 184-186). We also note that prior work, including our 2024 study in the NEJM, demonstrated increased IFN-γ signaling in ovarian tissue in Aire-deficient mice, providing supportive preclinical evidence for this mechanism. Accordingly, we now acknowledge that the mechanistic link in humans remains inferential in the absence of direct ovarian tissue data (Page 6, Line 260-265).

Moreover, we have added a statement emphasizing the need for longitudinal follow-up, including gynecological surveillance and assessment of fertility outcomes (Page 6, Lines 253-255).

Major Comment 4: *Transient asymptomatic EBV and BK viremia were detected in Patient 1 during a period of relative under-dosing (low CXCL9). The authors attribute this to immune reconstitution rather than immunosuppression, but this interpretation is not argued explicitly. The choice of acyclovir prophylaxis, which is inactive against BK virus, also needs justification. A structured viral surveillance protocol should be described. Given that this is a young child receiving JAK1/2 inhibition, this safety signal warrants a dedicated paragraph in the Discussion rather than a brief mention.*

Response: We thank the reviewer for this important point. We have clarified that the viremia occurred during a period of elevated CXCL9, consistent with relative underdosing of ruxolitinib (Page 5, Line 230-232). We have expanded the Discussion of the revised manuscript to more explicitly address the interpretation of these findings, including the distinction between primary infection and reactivation (Page 5, Line 232-235). We have also clarified the rationale for antiviral prophylaxis (targeting HSV and VZV) and now describe our structured viral surveillance approach, including periodic screening for BK, JC, CMV, and EBV viremia (Page 6, Line 235-236).

Major comment 5: *The proposal to use serum CXCL9 to guide ruxolitinib dosing is internally consistent and clinically interesting. However, the normal reference threshold used is not accompanied by a cited source or validated assay platform. Moreover, no formal correlation between CXCL9 and iPTH values over time in Patient 1 is provided. The authors should either present a Pearson or Spearman correlation across available longitudinal time points, or explicitly label this a pilot/exploratory observation pending validation in a larger cohort. Contextualizing CXCL9 within the existing literature supporting it as an IFN-gamma-dependent disease activity marker (e.g. in macrophage activation syndrome) would also help anchor this claim.*

Response: We thank the reviewer for these important points. We have clarified in the Methods that CXCL9 levels were measured in a CLIA-certified clinical laboratory using a validated microfluidics immunoassay (Page 7, Lines 297-301). We agree that the observations regarding CXCL9 as a biomarker of therapeutic response remain exploratory at this stage and have revised the manuscript accordingly, emphasizing the need for validation in larger cohorts. We have also further contextualized CXCL9 as an IFN-γ–dependent biomarker by referencing prior studies in IFN-γ–driven conditions such as hemophagocytic lymphohistiocytosis and macrophage activation syndrome (Page 6, Lines 241-246).

Minor Comment 1: *The claim that ruxolitinib achieves a "one-stop" multiorgan therapeutic effect would benefit from acknowledging that Hadjiyannis et al. (JACI 2025; DOI: 10.1016/j.jaci.2025.03.026) demonstrated comparable multiorgan remission with a selective JAK1 inhibitor. The Discussion should address whether JAK1 selectivity might offer a more favorable safety profile in the pediatric setting.*

Response: We thank the reviewer for this important point. We have revised the Discussion to acknowledge recent work demonstrating multiorgan remission with a JAK1-selective inhibitor. We note that JAK inhibitors exhibit relative, rather than absolute selectivity, and that additional data will be required to determine the comparative efficacy and safety of more selective inhibitors in APECED, particularly in pediatric populations (Page 5, Lines 213-218).

Minor Comment 2: *Weight gain as a potential pharmacokinetic confounder of mg/kg dosing is documented in Patient 1 but not discussed conceptually. The authors should recommend weight-based dose monitoring as a practical component of ruxolitinib management in growing children with APECED.*

Response: We agree that weight gain in growing children may affect mg/kg dosing and have revised the Discussion to emphasize the need for ongoing weight-based dose adjustment to maintain therapeutic exposure (Page 6, Lines 250-253).

Minor Comment 3: *Typographical error Line 141: “iPTH declied” should read “declined.”*

Response: This typographical error has now been corrected (Page 4, Line 153).

We thank the Editors and Reviewers for their thoughtful evaluation of our manuscript. We have addressed all comments and believe the revisions have strengthened the work. We appreciate the opportunity to submit this revised version for consideration and to disseminate these findings to the broader community.

Sincerely,

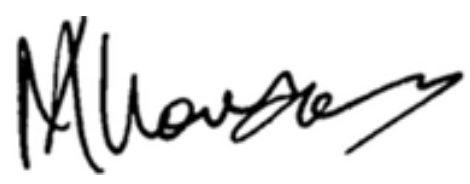

Michail S. Lionakis, MD, ScD
Senior Investigator
Chief, Fungal Pathogenesis Section
Chief, Laboratory of Clinical Immunology & Microbiology
National Institute of Allergy & Infectious Diseases (NIAID), NIH
