# Supplementary material for: Ruxolitinib prevents irreversible autoimmune endocrinopathies in APECED
Source: Nat Commun. 2026 Aug 1;17:9295. doi: 10.1038/s41467-026-76285-x (PMC13526882; doi:10.1038/s41467-026-76285-x)
Supplement: Supplementary file 3 [file 41467_2026_76285_MOESM3_ESM.pdf]

nature portfolio

Corresponding author(s): Michail S. Lionakis, MD, ScD

Last updated by author(s): 7/9/2026

Reporting Summary

Nature Portfolio wishes to improve the reproducibility of the work that we publish. This form provides structure for consistency and transparency in reporting. For further information on Nature Portfolio policies, see our Editorial Policies and the Editorial Policy Checklist.

Statistics

For all statistical analyses, confirm that the following items are present in the figure legend, table legend, main text, or Methods section.

n/a	Confirmed	
☐	☒	The exact sample size (*n*) for each experimental group/condition, given as a discrete number and unit of measurement
☐	☒	A statement on whether measurements were taken from distinct samples or whether the same sample was measured repeatedly
☐	☒	The statistical test(s) used AND whether they are one- or two-sided<br>*Only common tests should be described solely by name; describe more complex techniques in the Methods section.*
☐	☒	A description of all covariates tested
☐	☒	A description of any assumptions or corrections, such as tests of normality and adjustment for multiple comparisons
☐	☒	A full description of the statistical parameters including central tendency (e.g. means) or other basic estimates (e.g. regression coefficient) AND variation (e.g. standard deviation) or associated estimates of uncertainty (e.g. confidence intervals)
☐	☒	For null hypothesis testing, the test statistic (e.g. *F*, *t*, *r*) with confidence intervals, effect sizes, degrees of freedom and *P* value noted<br>*Give P values as exact values whenever suitable.*
☒	☐	For Bayesian analysis, information on the choice of priors and Markov chain Monte Carlo settings
☒	☐	For hierarchical and complex designs, identification of the appropriate level for tests and full reporting of outcomes
☒	☐	Estimates of effect sizes (e.g. Cohen's *d*, Pearson's *r*), indicating how they were calculated

Our web collection on statistics for biologists contains articles on many of the points above.

Software and code

Policy information about availability of computer code

Data collection	No software was used
Data analysis	PRISM 10 (version 10.2.0) was used for statistical analysis and graphic representation. NDP.view2 was used to capture representative images from histopathological slides.

For manuscripts utilizing custom algorithms or software that are central to the research but not yet described in published literature, software must be made available to editors and reviewers. We strongly encourage code deposition in a community repository (e.g. GitHub). See the Nature Portfolio guidelines for submitting code & software for further information.

Data

Policy information about availability of data

All manuscripts must include a data availability statement. This statement should provide the following information, where applicable:

- Accession codes, unique identifiers, or web links for publicly available datasets
- A description of any restrictions on data availability
- For clinical datasets or third party data, please ensure that the statement adheres to our policy

The authors attest that the main data supporting the findings of the study are available within the article and its Supplementary Information files.

Research involving human participants, their data, or biological material

Policy information about studies with human participants or human data. See also policy information about sex, gender (identity/presentation), and sexual orientation and race, ethnicity and racism.

Reporting on sex and gender	Given that this is a series involving only 2 patients, no sex or gender based analysis was possible. The general principles detailed here are not expected to apply to only one sex or gender noting that the reversal of hypogonadism detailed here is reviewed only in the context of a genetically female participant.
Reporting on race, ethnicity, or other socially relevant groupings	Given that this is a series involving only 2 patients, no race, ethnicity, or other socially relevant grouping analysis was performed.
Population characteristics	This is a case series of 2 patients with APECED. Patient 1 was a 2 year old male at the start of the events contained in this manuscript (with AIRE variants c.2T>A and c.967_979del in heterozygosity). Patient 2 was an 18 year old female (homozygous for the AIRE variant c.967_979del).
Recruitment	Participants were recruited from our natural history protocol (clinicaltrials.gov, NCT01386437). Patients were recruited to our natural history protocol by physician referral. As such, recruitment may be biased by referral patterns and toward participants with access to care.
Ethics oversight	Participants were enrolled on a natural history study overseen by the NIH IRB.

Note that full information on the approval of the study protocol must also be provided in the manuscript.

Field-specific reporting

Please select the one below that is the best fit for your research. If you are not sure, read the appropriate sections before making your selection.

[x] Life sciences [ ] Behavioural & social sciences [ ] Ecological, evolutionary & environmental sciences

For a reference copy of the document with all sections, see nature.com/documents/nr-reporting-summary-flat.pdf

Life sciences study design

All studies must disclose on these points even when the disclosure is negative.

Sample size	2 participants from a natural history protocol are included in this manuscript on the basis of identification of biochemical and/or clinical evidence of early endocrinopathy. The treatment and progress of these 2 patients is presented and thus no sample size calculations were performed.
Data exclusions	No data points were excluded from statistical analysis.
Replication	The assay in Figure 1E displays 3 technical replicates indicating successful replication. Other values presented are clinical outcomes and thus are without the possibility of replication.
Randomization	Treatment was open label in this 2 patient case series; as such, no randomization was performed.
Blinding	Treatment was open label in this 2 patient case series; as such, no blinding was performed.

Reporting for specific materials, systems and methods

We require information from authors about some types of materials, experimental systems and methods used in many studies. Here, indicate whether each material, system or method listed is relevant to your study. If you are not sure if a list item applies to your research, read the appropriate section before selecting a response.

Materials & experimental systems

n/a	Involved in the study	
[ ]	[x]	Antibodies
[x]	[ ]	Eukaryotic cell lines
[x]	[ ]	Palaeontology and archaeology
[x]	[ ]	Animals and other organisms
[ ]	[x]	Clinical data
[x]	[ ]	Dual use research of concern
[x]	[ ]	Plants

Methods

n/a	Involved in the study	
[x]	[ ]	ChIP-seq
[x]	[ ]	Flow cytometry
[x]	[ ]	MRI-based neuroimaging

Antibodies

Antibodies used	Anti-CD4 Rabbit Monoclonal Primary Antibody (Clone SP35, Roche, Cat No. 790-4423);<br>Anti-CD8 Rabbit Monoclonal Primary Antibody (Clone SP57, Roche, Cat No. 790-4460);<br>Anti-pSTAT1 (Clone 58D6, Cell Signaling Technology, Cat No. 9167)<br>All stains were performed in a CLIA certified pathology lab using antibodies validated for use on the clinical service.
Validation	Anti-CD4: Recommended for IHC; cited in 124 publications; Additional documentation on manufacturer's website: https://diagnostics.roche.com/us/en/products/lab/cd4-sp35-confirm-rtd000770.html#documents<br><br>Anti-CD8: Recommended for IHC; no further data available on manufacturer's website<br><br>Anti-pSTAT1: Recommended for IHC; cited in 1424 publications; additional information on manufacturer's website: https://www.cellsignal.com/products/primary-antibodies/phospho-stat1-tyr701-58d6-rabbit-monoclonal-antibody/9167?srsltid=AfmBOop1XcKCgPJx8e3sOHLuPAeEZrqDtIY6Ay0sbeD8-AFNftvHw-8h)

Clinical data

Policy information about clinical studies

All manuscripts should comply with the ICMJE guidelines for publication of clinical research and a completed CONSORT checklist must be included with all submissions.

Clinical trial registration	clinicaltrials.gov, NCT01386437
Study protocol	The full protocol is available on clinicaltrials.gov
Data collection	Patient 1 was recruited to our study in 2023 while Patient 2 was in 2016. The events of this manuscript occurred between 2023 and March 2026. All data on Patient 1 was collected either at the NIH Clinical Center in Bethesda, MD or at CHOP in Philadelphia, PA. Data on Patient 2 was collected either at the NIH Clinical Center in Bethesda, MD or by her physician at University College Dublin in Dublin, Ireland.
Outcomes	Our primary outcomes were selected as clinical and biochemical reversal of early autoimmune endocrinopathies in 2 patients (hypoparathyroidism in Patient 1 and hypogonadism in Patient 2). Biochemical readouts of hypoparathyroidism (iPTH, serum calcium, ionized calcium, serum phosphate) and hypogonadism (FSH, LH, AMH, estradiol) were measured in a CLIA certified clinical laboratory. Safety endpoints for Patient 1 presented here (HGB, PLT) were measured in a CLIA certified clinical lab or (height, weight) were assessed by qualified personnel on clinic visits.

Plants

Seed stocks	N/A
Novel plant genotypes	N/A
Authentication	N/A
